# Stable isotope turnover rates and fractionation in captive California yellowtail (*Seriola dorsalis*): insights for application to field studies

**DOI:** 10.1038/s41598-021-83880-z

**Published:** 2021-02-24

**Authors:** Daniel J. Madigan, Owyn E. Snodgrass, John R. Hyde, Heidi Dewar

**Affiliations:** 1 Department of Organismic and Evolutionary Biology, Harvard University, Cambridge, MA 02138 USA; 2 Department of Integrative Biology, University of Windsor, Windsor, ON N9B 3P4 Canada; 3 Fisheries Resources Division, Southwest Fisheries Science Center, National Marine Fisheries Service (NMFS), National Oceanic and Atmospheric Administration (NOAA), La Jolla, CA 92037 USA

**Keywords:** Animal migration, Stable isotope analysis, Ecosystem ecology

## Abstract

Stable isotope analysis (SIA) measurements from long-term captivity studies provide required parameters for interpretation of consumer SIA data. We raised young-of-the-year (14–19 cm) California yellowtail (*Seriola dorsalis*) on a low δ^15^N and δ^13^C diet (pellet aquaculture feed) for 525 days, then switched to a high δ^15^N and δ^13^C diet (mackerel and squid) for 753 days. Yellowtail muscle was sequentially sampled from each individual after the diet switch (0 to 753 days) and analyzed for δ^15^N and δ^13^C, allowing for calculation of diet-tissue discrimination factors (DTDFs) from two isotopically different diets (low δ^15^N and δ^13^C: pellets; high δ^15^N and δ^13^C: fish/squid) and turnover rates of ^15^N and ^13^C. DTDFs were diet dependent: Δ^15^N = 5.1‰, Δ^13^C = 3.6‰ for pellets and Δ^15^N = 2.6‰, Δ^13^C = 1.3‰ for fish/squid. Half-life estimates from ^15^N and ^13^C turnover rates for pooled yellowtail were 181 days and 341 days, respectively, but varied considerably by individual (^15^N: 99–239 d; ^13^C: 158–899 d). Quantifying DTDFs supports isotopic approaches to field data that assume isotopic steady-state conditions (*e.g*., mixing models for diet reconstruction). Characterizing and quantifying turnover rates allow for estimates of diet/habitat shifts and “isotopic clock” approaches, and observed inter-individual variability suggests the need for large datasets in field studies. We provide diet-dependent DTDFs and growth effects on turnover rates, and associated error around these parameters, for application to field-collected SIA data from other large teleosts.

## Introduction

Fundamental understanding of fish biology, feeding behavior, population dynamics, and movement patterns has expanded in recent years due to technological advances in tools such as electronic tags for animal tracking^1,2^, chemical tracer analyses^3^, and ‘big data’ analysis^4,5^. However, knowledge gaps remain for all fish species^6,7^, which can be addressed with evolving tools for more comprehensive, ecosystem-based understanding and management of fish species. Stable isotope analysis (SIA) is one tool that has rapidly expanded in marine ecosystem studies. Ecological studies using SIA rely on the fact that marine consumers reflect the isotopic composition of the prey they consume, which varies with prey ecology, region, and environmental conditions^8,9^. This premise has allowed researchers to investigate diet, trophic dynamics, and movements of marine predators^8,10,11^ with SIA, most commonly of nitrogen (δ^15^N) and carbon (δ^13^C). More recently, the quantity of SIA data for marine predators has grown to allow for global analyses of marine predator ecology and habitat use. For example, the synthesis of thousands of SIA values across ocean basins has allowed for characterization of global trophic geography in sharks^12^ and tunas^13^.

Underlying consumer SIA data are complex physiological and ecological isotopic dynamics, which ultimately determine consumer SI values. Isotopic compositions of consumer tissues reflect those of diet, which guided early applications of SIA to feeding ecology. Consumer tissue δ^15^N and δ^13^C increase systematically during trophic transfer due to fractionation and re-routing dynamics of amino acids and other organic compounds during digestion, assimilation, and tissue turnover^14^. The resultant differences between consumer and diet SIA values, represented by several equivalent terms (diet-tissue discrimination factors [DTDFs], trophic discrimination factors [TDFs], or trophic enrichment factors [TEFs]), have been used for trophic level estimates and food web reconstruction. DTDF estimates for δ^15^N (Δ^15^N) have generally been higher than those for δ^13^C (Δ^13^C), and an early synthesis of DTDF values reported a widely used, multi-taxon mean of 3.4 (± 1.0 SD) for Δ^15^N and 0.4 (± 1.3 SD) for Δ^13^C^10^. Many early ecological studies applied these first multi-taxon DTDF estimates to estimate diet and trophic position, but subsequent laboratory studies have provided many more taxon- or species-specific DTDFs, demonstrating the importance of applying specific DTDF values due to substantial variability across taxa, tissue types, and diet^14,15^. While statistical frameworks and software packages that reconstruct predator diets using SIA (e.g. MixSIR, SIAR)^16,17^ are able to incorporate error around DTDF values^18^, mixing models are better parameterized by laboratory studies that provide more accurate DTDF estimates^19^.

Understanding isotopic turnover rates in predator tissues is required to quantify timing of diet shifts or animal movements. Following a shift to an isotopically different diet, predator tissue δ^15^N and δ^13^C values change over time until reaching consistent values that reflect the new diet (i.e., steady-state conditions). These rates are largely dictated by the physiological processes of new tissue synthesis (growth) and anabolic and catabolic ‘tissue turnover’ processes (broadly referred to here as ‘metabolism’). Captive studies in fish have shown muscle isotopic turnover rates as low as several days (young-of-the-year [YOY] winter flounder *Pseudopleuronectes americanus*^20^) to months (summer flounder *Paralichthys dentatus*, arctic sculpin *Myoxocephalus scorpioides*^21,22^) to > 1 year (mado *Atypichthys latus*, Pacific bluefin tuna *Thunnus orientalis*^23,24^). These differences are largely driven by fish size and life-stage; since relative growth (proportion of new mass to initial mass over time) is a primary driver of isotopic turnover dynamics, turnover rates will vary with relative growth rates ontogenetically (faster turnover in younger fish) and inter-specifically (faster turnover in fish with high growth rates). Isotopic turnover rates have most often been described with exponential fit models, allowing for calculation of half-life (*t*_0.5_) values for comparison across studies^25–27^. Recent reviews and meta-analyses of laboratory-derived isotopic turnover rates have reported predictable allometric relationships of slower turnover rates (higher *t*_0.5_) with increasing fish body size^25–27^. Turnover rates are also tissue-dependent; more metabolically active ‘fast-turnover’ tissues (e.g. blood plasma, liver) and ‘slow-turnover’ tissues (e.g. muscle, bone collagen) can reflect a shift in diet of large predatory fish in timeframes of days/weeks or months/years, respectively^28^.

Laboratory-based experiments, under controlled or semi-controlled conditions, quantifying species- and lifestage-specific isotopic turnover improve the understanding of stable isotope dynamics and have direct applications to wild SIA studies. Turnover rates and the time to steady-state allow researchers to put diet estimates in temporal context by roughly demonstrating the dietary timeframes represented by SIA composition of various tissues. Quantified turnover rates also allow applied ‘isotopic clock’ techniques^29–32^, which use isotopic endmembers (of predator, diet, and/or region) and measured predator SIA values to estimate the timing since a shift in diet and/or habitat. Measured SIA values in fish that recently migrated thus allow retrospective reconstructions of large-scale movements, such as entire river migrations by lake sturgeon (*Acipenser fulvescens*)^33^ and trans-Pacific migrations by Pacific bluefin tuna^34^. However, accuracy of these migratory timeframes is maximized with accurate species-specific isotopic turnover rates, which are best calculated from laboratory experiments.

**Figure 1:**
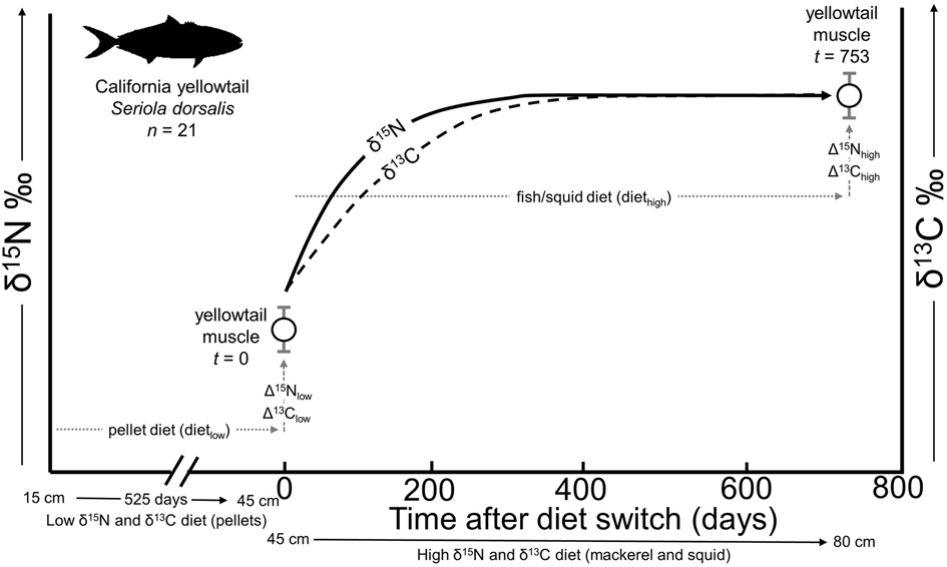
Schematic showing experimental design to quantify dynamics of δ^15^N and δ^13^C in captive California yellowtail *Seriola dorsalis*. Sizes (cm) below x-axis show approximate changes in yellowtail fork length during the phases of the experiment. Lines showing changes in yellowtail muscle δ^15^N (solid line) and δ^13^C (dashed line) are theoretical changes based on typical isotopic turnover dynamics in teleosts.

The California yellowtail (*Seriola dorsalis*) is an ecologically and economically important teleost predator in the California Current Ecosystem. At typical capture sizes (40 cm to > 100 cm), yellowtail are a relatively large-sized, highly mobile, high trophic level predator in pelagic and coastal ecosystems^35^. The lack of experimentally-derived DTDFs and turnover parameters for yellowtail limits the interpretation of SIA data for (1) temporal and spatial patterns in trophic dynamics and (2) timing of movement patterns, as yellowtail are known to shift from pelagic to coastal habitats with increasing size^36,37^. To calculate these isotopic parameters for this species, we raised YOY yellowtail to steady-state on a low δ^15^N and δ^13^C diet for 525 days, then switched larger (~ 40 cm) yellowtail to a high δ^15^N and δ^13^C diet and sequentially sampled muscle from individuals over 753 days (see Fig. 1). This allowed for quantification of DTDFs in yellowtail, as well as isotopic turnover in individuals and across the study population. These isotopic parameters can be applied to field-collected SIA data for active, high trophic level teleosts that currently lack laboratory-derived SIA data.

## Results

### Yellowtail diet δ^15^N and δ^13^C values

The δ^15^N and δ^13^C values of pellet feed (n = 10) were 5.8 ± 0.1‰ SD and  − 22.6 ± 0.4‰, respectively. The δ^15^N and δ^13^C values of Pacific mackerel (n = 6) and market squid (n = 6) were (mackerel) 13.5 ± 0.0‰ and  − 19.7 ± 0.1‰ and (squid) 14.7 ± 0.3‰ and  − 17.9 ± 0.5‰, resulting in weighted mean fish/squid (weighted by % fish and % squid presented to yellowtail; 68% and 32% respectively) diet δ^15^N and δ^13^C values of 14.0 ± 0.8‰ and  − 19.0 ± 0.9‰ (Table 1).

**Table 1:** Mean δ^15^N, δ^13^C, and C:N ratio values of California yellowtail (*Seriola dorsalis*) muscle tissue, captive feed, and calculated diet-specific DTDF values for pellet and fish/squid diet.

Group	Isotope	Tissue	n	Mean (SD)	C:N (SD) (mass)			
Feed								
Pellets	δ^13^C	–	10	− 22.6 (0.4)	7.0 (0.5)			
	δ^15^N	–	10	5.8 (0.1)	7.0 (0.5)			
Mackerel	δ^13^C	WM	6	− 19.7 (0.1)	3.2 (0)			
	δ^15^N	WM	6	13.5 (0.1)	3.2 (0)			
Squid	δ^13^C	WM	6	− 17.9 (0.5)	3.4 (0)			
	δ^15^N	WM	6	14.7 (0.3)	3.4 (0)			
Fish/squid mean	δ^13^C	WM	12	− 19.0 (0.9)	–			
Weighted by mass	δ^15^N	WM	12	14.0 (0.8)	–			

Yellowtail						DTDF		
						Mean	SD	Time in capt
Diet_low_	*δ*^15^N	WM	21	10.9 (0.3)	3.6 (0.3)	5.1	0.3	525 d
Diet_low_*	*δ*^13^C	WM	21	− 19.0 (0.1)	3.6 (0.3)	3.6*	0.1	525 d
Diet_high_	*δ*^15^N	WM	10	16.5 (0.2)	3.4 (0.2)	2.6	0.2	> 595 d
Diet_high_	*δ*^13^C	WM	10	− 17.7 (0.4)	3.4 (0.2)	1.3	0.4	> 595 d

δ^13^C values are arithmetically lipid-corrected according to Logan et al.^55^.

*The δ^13^C values of Diet_low_ (pellet diet) could not be corrected for carbonate and or lipid due to unknown pellet composition; this DTDF is based on bulk pellet δ^13^C values.

### Diet-tissue discrimination factors

We calculated two DTDF values for δ^15^N and δ^13^C (Δ^15^N and Δ^13^C) from captive yellowtail due to the demonstrated correlation between fish DTDF values and diet δ^15^N and δ^13^C values (Caut et al. 2009). The two diets used here differed highly in δ^15^N and δ^13^C values (Table 1). For YOY yellowtail fed pellet feed (low δ^15^N and δ^13^C diet) for 525 d, Δ^15^N_low_ and Δ^13^C_low_ were 5.1 ± 0.3‰ and 3.6 ± 0.1‰, respectively (n = 21) (Table 1). Individual yellowtail Δ^15^N_low_ values ranged from 3.4–3.9‰ and Δ^13^C_low_ values from 4.6–5.7‰. For larger yellowtail fed fish/squid (high δ^15^N and δ^13^C diet) for 595–753 d, Δ^15^N_high_ and Δ^13^C_high_ were 2.6 ± 0.2‰ and 1.3 ± 0.4‰, respectively (Table 1). Individual yellowtail Δ^15^N_high_ values ranged from 2.3–2.9‰ and Δ^13^C_high_ values from 0.7–2.0‰.

We compared the observed relationship between DTDF and diet δ^15^N and δ^13^C values with relationships quantified previously in fishes by Caut et al. (2009) and Hussey et al. (2014). We used experimental diet δ^15^N and δ^13^C values to calculate estimates of Δ^15^N_low_ and Δ^13^C_low_ and Δ^15^N_high_ and Δ^13^C_high_ from Caut et al. (2009) (Eqs. 6, 7), and of Δ^15^N_low_ and Δ^15^N_high_ from Hussey et al. (2014) which only quantified the relationship for Δ^15^N (Eq. 8)_._ Calculated estimates were: Δ^15^N_low_ = 4.2‰; Δ^13^C_low_ = 2.0‰; Δ^15^N_high_ = 2.1‰; Δ^13^C_high_ = 1.2‰ (Caut et al. 2009); ^15^N_low_ = 4.4‰; Δ^15^N_high_ = 2.2‰ (Hussey et al. 2014). By modifying the constants in Eqs. 6 and 7 (Caut et al. 2009) to fit results here, we provide predictive equations for DTDFs of yellowtail based on diet δ^15^N and δ^13^C values:

1 $$\Delta^{15} {\text{N}}_{{{\text{yellowtail}}}} = \, - 0.317(\delta^{15} {\text{N}}_{{{\text{diet}}}} ) \, + \, 6.939$$

2 $$\Delta^{13} {\text{C}}_{{{\text{yellowtail}}}} = \, - 0.639(\delta^{13} {\text{C}}_{{{\text{diet}}}} ) \, {-} \, 10.839$$

### Time-based δ^15^N and δ^13^C turnover

**Figure 2:**
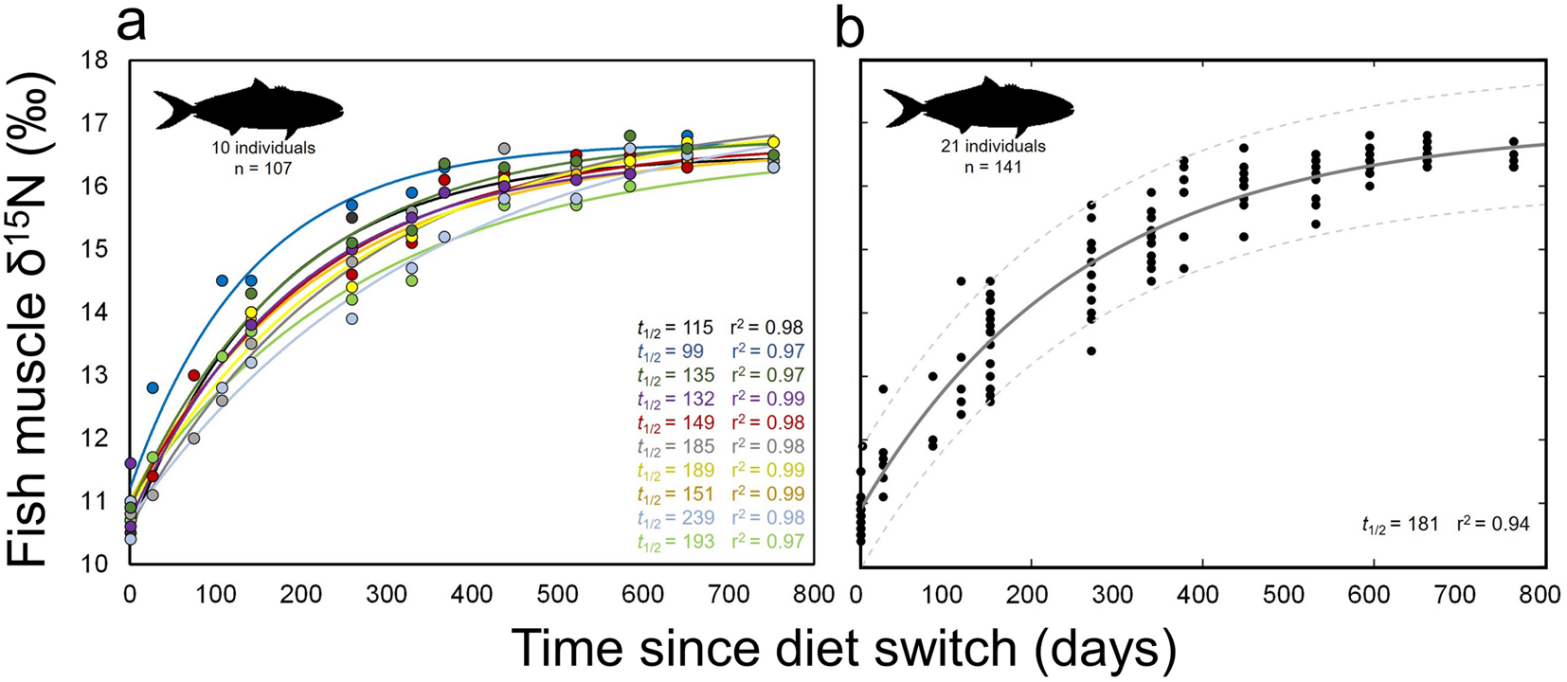
Change of δ^15^N with time after diet switch in muscle of (**a**) individual and (**b**) grouped captive California yellowtail *Seriola dorsalis*. (**a**) Points represent individual δ^15^N measurements and lines show associated model fits, colored by individual. Values *t*_1/2_ is the half-life of ^15^N for each individual and r^2^ values are shown for model fit to each individual. Data shown only for yellowtail that were in captive conditions for enough time to reach steady-state with new diet. (**b**) Model fit (solid line) and 95% confidence bounds (dashed lines) for all yellowtail sampled. Grouped data include fish that were removed from captivity or suffered mortality during the experimental period.

Pooled yellowtail δ^15^N values increased from 0–378 d, increasing from 10.9 ± 0.3‰ to 15.8 ± 0.6‰ over this period (Fig. 2, Table 2). After 378 d, time-based increases in δ^15^N were lower, reaching apparent steady-state at 595 d at 16.4 ± 0.2‰ (Fig. 2). δ^15^N values then remained consistent to 753 d (16.4 ± 0.2‰). The exponential model fit grouped yellowtail data well (r^2^ = 0.94; Fig. 2) and provided a time-based estimate of ^15^N *t*_0.5_ of 181 d (Fig. 2b) (see Table 3 for model parameters and 95% confidence intervals).

**Table 2:** Mean stable isotope values and time in captivity for all California yellowtail (*Seriola dorsalis*) used in this study.

n	Time in captivity (d)	WM *δ*^15^N (SD)	WM *δ*^13^C (SD)
21	0	10.9 (0.3)	− 19.0 (0.1)
9	1–99	11.9 (0.6)	− 18.8 (0.2)
22	100–199	13.5 (0.7)	− 18.4 (0.2)
12	200–299	14.6 (0.7)	− 18.2 (0.2)
21	300–399	15.4 (0.6)	− 18.0 (0.2)
12	400–499	16.0 (0.5)	− 17.7 (0.2)
20	500–599	16.3 (0.3)	− 17.6 (0.2)
10	600–700	16.5 (0.2)	− 17.4 (0.2)
10	753	16.4 (0.2)	− 17.5 (0.1)

*δ*^13^C values are arithmetically-corrected for lipid content based on tissue-specific (fish muscle) algorithms from Logan et al^55^. Note not all fish could be sampled at every sampling period, resulting in varying sample size (n) across the experimental period.

**Table 3:** Parameter estimates and calculated turnover rate metrics from time-based exponential fit models for δ^15^N and δ^13^C values of muscle in California yellowtail (*Seriola dorsalis*).

Isotope	Parameter (95% CI)			*r*^2^	*t*_0.5_ (d) (95% CI)	*t*_0.95_ (d)
	*a*	*λ*	*c*			
δ^15^N	− 6.0 (−6.4, -5.7)	0.0038 (0.0033, 0.0043)	16.9 (16.6, 17.2)	0.94	181 (160, 210)	784
δ^13^C	− 2.0 (−2.3, -1.7)	0.0020 (0.0015, 0.0026)	− 16.9 (−17.3, −16.6)	0.91	341 (265, 478)	1473

Estimated half-life (*t*_0.5_) and time for 95% isotope turnover (*t*_0.95_) (days) is shown for each isotope.

Individual yellowtail δ^15^N turnover rates were variable, with a range of individual *t*_0.5_ values of 99–239 d (Fig. 2a). All individuals reached consistent, steady-state values by 595 d, and high individual *t*_0.5_ values were driven by relatively low δ^15^N values at intermediate timesteps, resulting in exponential model fits that reached asymptotic steady-state values late in the experiment for some individual fish (Fig. 2a). However, intra-individual turnover dynamics were consistent with first-order exponential model predictions, with high exponential model fits across individual yellowtail (*r*^2^ range 0.97–0.99, mean 0.98 ± 0.1; Fig. 2a) and higher *r*^2^ values for all individuals than for the single model fit across all yellowtail (*r*^2^ = 0.94; Fig. 2b).

**Figure 3:**
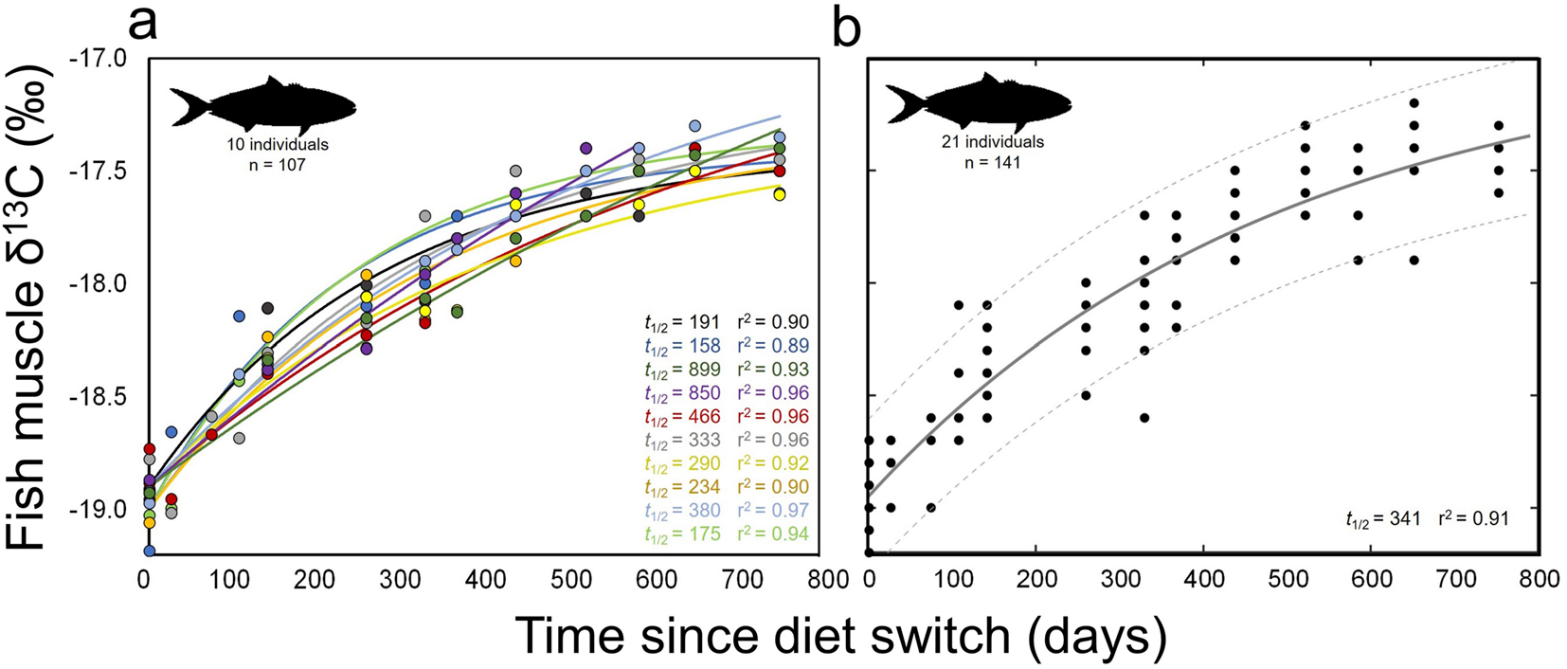
Change of δ^13^C with time after diet switch in muscle of (**a**) individual and (**b**) grouped captive California yellowtail *Seriola dorsalis*. (**a**) Points represent individual δ^13^C measurements and lines show associated model fits, colored by individual. Values *t*_1/2_ is the half-life of ^13^C for each individual and *r*^2^ values are shown for model fit to each individual. Data shown only for yellowtail that were in captive conditions for enough time to reach steady-state with new diet. (**b**) Model fit (solid line) and 95% confidence bounds (dashed lines) for all yellowtail sampled. Grouped data includes fish that were removed from captivity or suffered mortality during the experimental period.

Pooled yellowtail muscle δ^13^C values also increased consistently throughout the experiment (Fig. 3 and Table 2). δ^13^C values increased from 0–532 days, increasing from -19.0 ± 0.1‰ to  − 17.6 ± 0.2‰ over this period (Fig. 3). Subsequent δ^13^C values were generally consistent, reaching  − 17.5 ± 0.1‰ at 753 d. Model fits showed an asymptote at longer time periods than for δ^15^N both for individual yellowtail (Fig. 3a) and grouped data (Fig. 3b). The exponential model fit grouped yellowtail data well (*r*^2^ = 0.91; Fig. 3b and Table 3) and provided a time-based estimate of ^13^C *t*_0.5_ of 341 d (Fig. 3b; see Table 3 for model parameters and 95% confidence intervals).

Across individual yellowtail, turnover rates of δ^13^C were more variable than those of δ^15^N, with a range of individual δ^13^C *t*_0.5_ values of 158–899 d (Fig. 3a). All individuals reached steady-state values by the end of the experiment, though the dynamics of δ^13^C change at earlier timesteps resulted in exponential model fits that reached asymptotic steady-state values late in the experiment, or not at all, in individual fish (Fig. 3a). As for δ^15^N, intra-individual turnover of δ^13^C was fairly consistent with the first-order exponential model (*r*^2^ range 0.89–0.97, mean 0.93 ± 0.3; Fig. 3a); most individual model fits were better than the single model fit across all yellowtail (*r*^2^ = 0.91; Fig. 3b).

### Growth-based δ^13^C and δ^15^N turnover

Yellowtail that remained in captive conditions for adequate time periods for individual turnover estimates (*n* = 10) showed substantial but variable growth in captivity. Relative growth in mass *W*_*R*_ (see Eq. 13) by the end of the experimental period (753 d) ranged from 1.7–3.5 (mean 2.7 ± 0.5 SD). Growth rate constants for individual fish ranged from 0.0008 to 0.0023 d^−1^ (0.0017 ± 0.0005 d^−1^). The model-estimated growth rate constant (*k*ʹ; see Eq. 15) for the grouped population of yellowtail (0.0017 d^−1^) was the same as the mean across individuals (0.0017 ± 0.0005 d^−1^).

**Figure 4:**
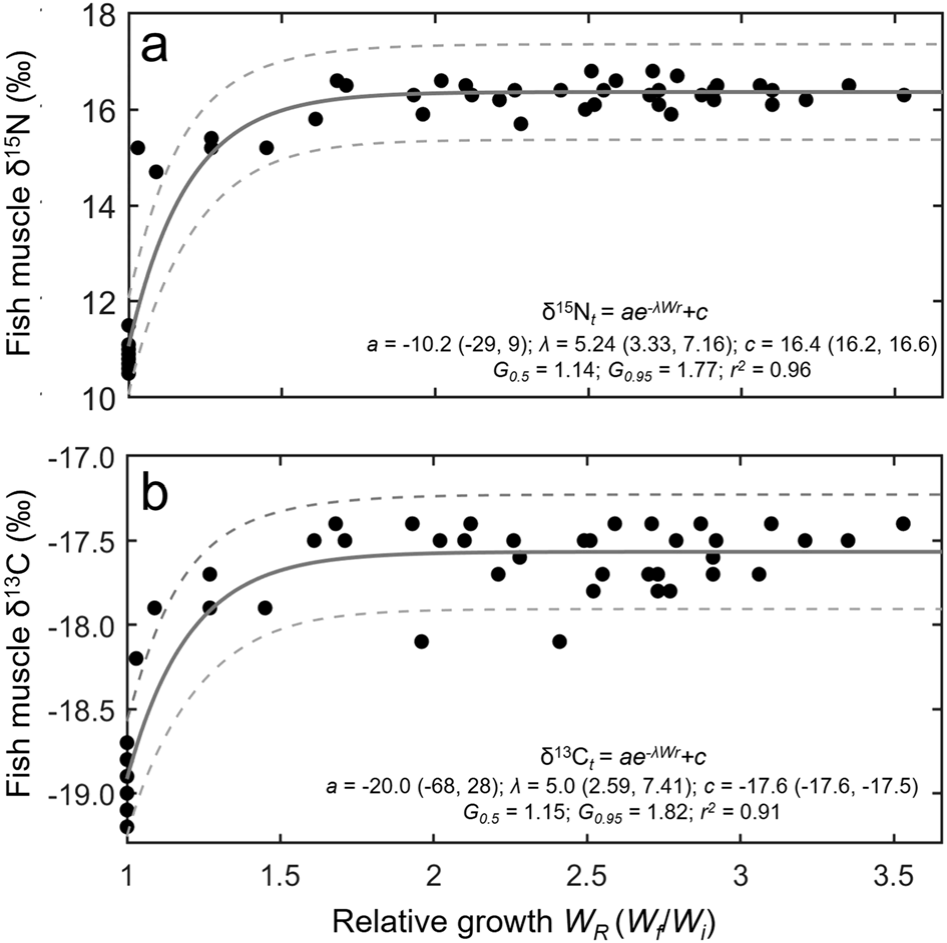
Isotopic change with fish growth for (**a**) δ^15^N and (**b**) δ^13^C in captive California yellowtail *Seriola dorsalis.* Points represent individual measurements and lines show associated model fits (solid lines) and 95% confidence intervals (dashed lines). Model equation and associated parameters (95% confidence intervals) shown for both isotopes. *G*_0.5_ and G_0.95_ show the amount of relative growth by mass (mass_final_/mass_initial_) required for 50% turnover and 95% turnover, respectively, of each isotope in yellowtail muscle.

Change in yellowtail δ^15^N and δ^13^C values were well described by exponential model fits to *W*_*R*_ (Fig. 4, Table 4). Model fit of *W*_*R*_ to change in δ^15^N was slightly better (*r*^2^ = 0.96) than that for δ^13^C (*r*^2^ = 0.91) (Fig. 4, Table 4). For δ^15^N, the relative growth in mass required for 50% turnover and 95% turnover was 1.14 and 1.77, respectively (Fig. 4a, Table 4). For δ^13^C, the *W*_*R*_ required for 50% turnover and 95% turnover was 1.15 and 1.82, respectively (Fig. 4b, Table 4).

**Table 4:** Parameter estimates and 95% confidence intervals for relative growth-based (*W*_*R*_) exponential fit models for δ^15^N and δ^13^C values of muscle in California yellowtail (*Seriola dorsalis*).

Isotope	Parameter (95% CI)			*r*^2^	*G*_0.5_	*G*_0.95_
	*a*	*λ*	*c*			
δ^15^N	− 10.2 (−29.1, 9.1)	5.242 (3.327, 7.156)	16.4 (16.2, 16.6)	0.96	1.14	1.77
δ^13^C	− 20 (−68, 28)	5.0 (2.590, 7.407)	− 17.6 (−17.6, −17.5)	0.91	1.15	1.82

Estimated growth-based half-life (*G*_0.5_) and growth required for 95% isotopic turnover (*G*_0.95_) is shown for each isotope.

### Relative proportion of isotopic turnover due to growth and metabolic processes

**Figure 5:**
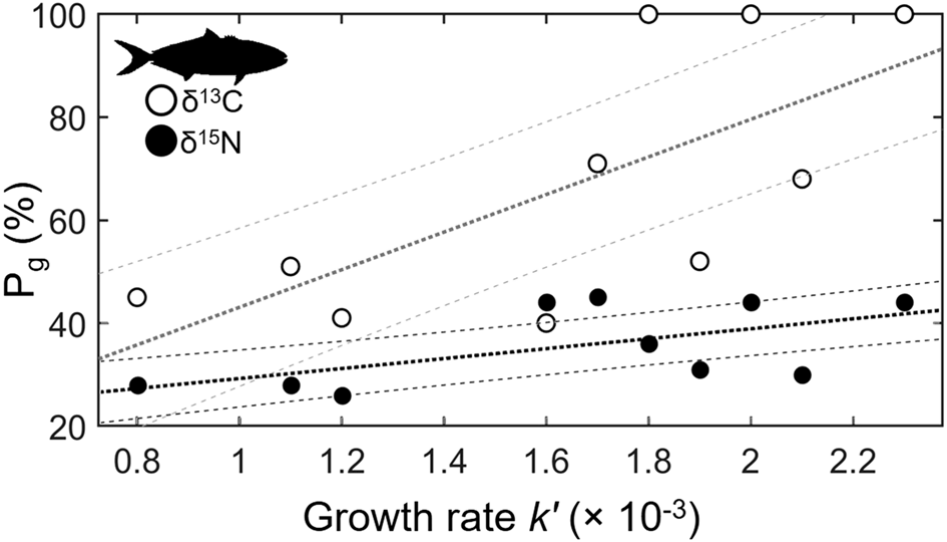
Proportion of isotopic turnover in muscle due to growth (P_g_) increases with growth rate in yellowtail *Seriola dorsalis.* Points represent individual measurements of δ^15^N (filled circles) and δ^13^C (open circles) and lines show associated linear fits (dark dashed lines) and 95% confidence intervals (light dashed lines). Positive relationships were significant between *P*_*g*_ and *k*ʹ for both δ^13^C (linear regression, *P* = 0.03) and δ^15^N (linear regression, *P* = 0.02).

Sequential sampling of individual yellowtail allowed for calculation of the proportion of isotopic turnover due to growth (*P*_*g*_) and proportion of turnover due to metabolic processes (*P*_*m*_) (see Eqs. 19, 20) across individual fish and the grouped population of captive fish. Overall, growth contributed more to turnover of ^13^C (*P*_*g*_ = 84%, *P*_*m*_ = 16%) than ^15^N (*P*_*g*_ = 44%, *P*_*m*_ = 56%) for the grouped population of captive yellowtail. These proportional estimates varied in individual yellowtail. *P*_*g*_ for turnover of ^15^N ranged from 26 to 45% (36 ± 8%), and *P*_*g*_ for turnover of ^13^C ranged from 40 to 100% (65 ± 26%). Metabolic processes *P*_*m*_ contributed to the majority of ^15^N turnover in all fish (55–74%, 64 ± 8%). For ^13^C turnover, *P*_*m*_ was more variable, contributing 0% to several fish, but to the slight majority (51–60%) of ^13^C turnover in 4 of 10 fish (0–60%, 35 ± 26%). Growth rate *k*ʹ of individual fish was significantly correlated with *P*_*g*_ for turnover of both isotopes, with faster growth rates resulting in higher proportion of turnover due to growth for ^15^N (linear regression, *P* = 0.02) and ^13^C (linear regression, *P* = 0.03) (Fig. 5).

### Applicability to field data

**Figure 6:**
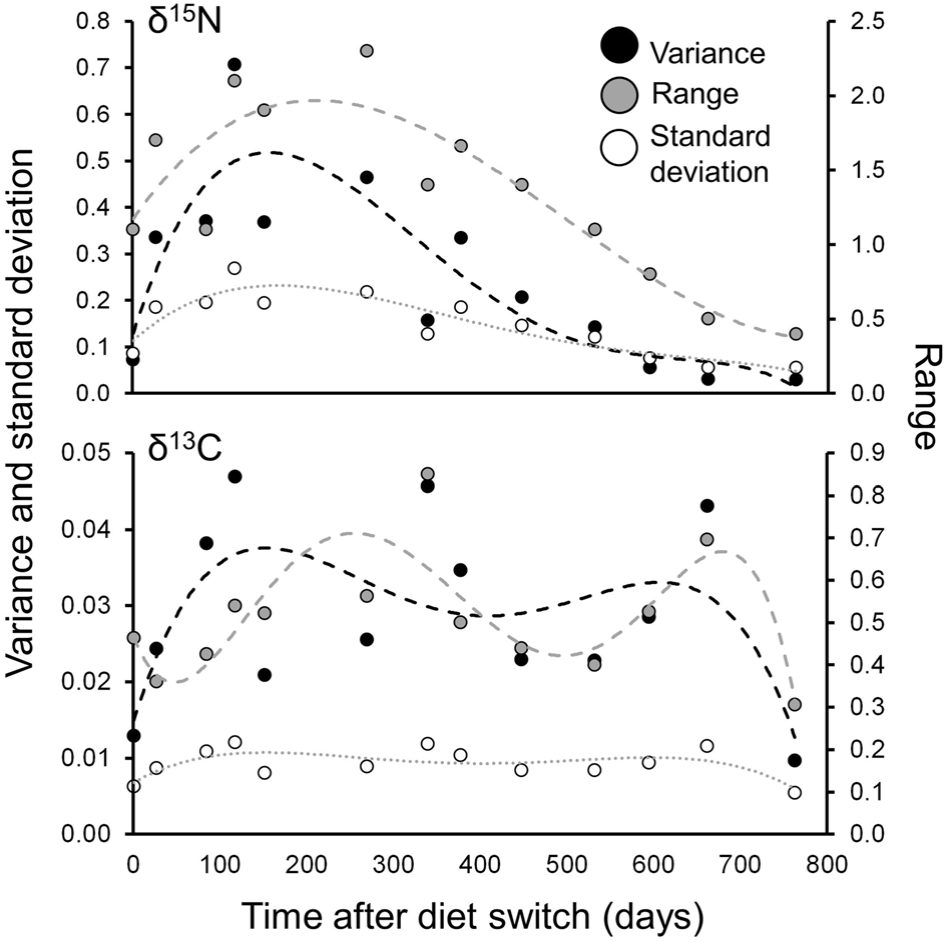
Variability of muscle δ^15^N and δ^13^C with time across individual California yellowtail *Seriola dorsalis* following a diet switch in captivity*.* Points represent individual measurements and dashed lines show associated polynomial model fits (solid lines) for various metrics of variability: variance (black), range (grey) and SD (white). Note that through the experimental period, variability was lowest at the beginning and end, representing steady-state with low δ^15^N and δ^13^C diet (pellets) and high δ^15^N and δ^13^C diet (fish/squid), respectively.

Based on three metrics (variance, range, standard deviation), yellowtail muscle δ^15^N values showed the lowest variability at the beginning (t = 0 d) and end (t = 753 d) of the diet switch experiment (Fig. 6). Variability rapidly increased after the diet switch and was highest between 100–400 d (Fig. 6). These temporal patterns of variability were less evident for δ^13^C, but δ^13^C values also were generally most consistent at the beginning and end of the experimental period (Fig. 6).

**Figure 7:**
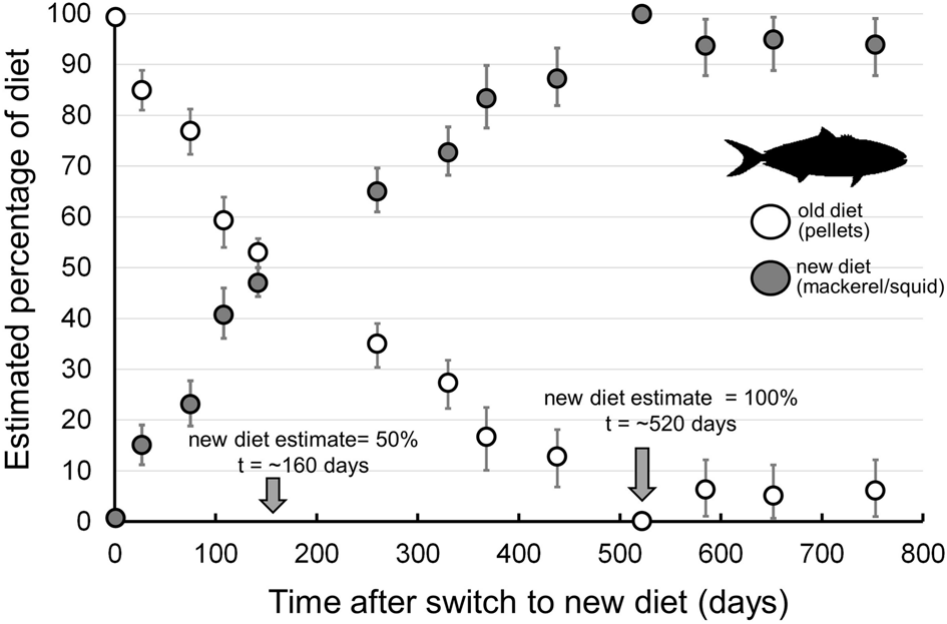
Bayesian mixing model diet estimates over time in captive California yellowtail *Seriola dorsalis*, showing time (days) required for mixing models to accurately reflect diet switch in yellowtail muscle*.* Points represent median diet proportion estimates and error bars show 95% credible intervals. Arrows show the approximate time for yellowtail muscle to represent 50% new diet and 100% new diet. Note that ~ 520 days were required for mixing model estimates to correctly show 100% of new diet, representing ~ 3 half-lives for ^15^N (181 d) and ~ 1.5 half-lives for ^13^C (341 d), as calculated from turnover rates in this experiment.

At the start of the diet switch, when yellowtail began feeding on 100% fish/squid, Bayesian mixing model estimates showed 100% pellet diet at *t* = 0 (Fig. 7). After ~ 160 days of fish/squid diet, yellowtail muscle δ^15^N and δ^13^C values resulted in mixing model estimates of 50% pellets, 50% fish/squid (Fig. 7). Approximately 520 days were required for mixing model estimates to reflect 100% fish/squid diet (Fig. 7), representing ~ 3 ^15^N half-life periods and ~ 1.5 ^13^C half-life periods.

## Discussion

Species-specific turnover experiments provide crucial parameters for application to stable isotope ecology studies. We reared a large teleost in semi-controlled conditions (here, controlled diet with fluctuating temperature and salinity) to provide DTDFs and isotopic turnover estimates, which are relatively rare due to the difficulty of large fish husbandry and the timeframes required for such experiments. We demonstrate that DTDFs are diet-dependent in California yellowtail, similar to other fish reared on similar diets, and provide DTDF values and predictive algorithms that allow for diet-dependent discrimination factors to be estimated for active large-bodied teleosts. The relatively long turnover rates reported here (steady-state conditions after > 1 yr) demonstrate the long timeframes required for wild fish to reach steady-state conditions with local diet. Finally, turnover rates provide parameters for field-based estimates of diet shifts and movements, and growth-based models allow for scaling of reported turnover rates with animal size and associated changes in relative growth rates.

Yellowtail Δ^15^N and Δ^13^C estimates were strongly influenced by diet. Δ^15^N and Δ^13^C were 5.1‰ and 3.6‰ for low δ^15^N and δ^13^C diet (pellets) and 2.5‰ and 1.3‰ for high δ^15^N and δ^13^C diet (fish/squid). Our Δ^15^N and Δ^13^C values are different from means that have been reported in DTDF reviews: (Δ^15^N) 3.4‰^10,38^, 3.2‰^39^, 2.9‰^40^, 2.5‰^41^, and 2.0‰^42^ and (Δ^13^C) 0.4‰^10,42^ and 0.5‰^40^. However, taxon- and species specific DTDFs have proven to be broad, and our values fit within the wide ranges of specific DTDFs reported across reviewed studies (Δ^15^N =  − 3.2–9.2‰ and Δ^13^C =  − 8.8–6.1‰^14^). Δ^13^C values typically have been described as low, citing minimal fractionation of ^13^C between trophic levels and thus being useful as tracking the source of productivity in the diet of upper trophic level consumers. However, our Δ^13^C values of 3.6‰ and 1.3‰ suggest a much higher degree of fractionation of ^13^C. However, our calculated Δ^13^C_low_, from pellet diet, should be interpreted with caution as pellet composition was unknown, pellet C:N was high (7.0 ± 0.5), and unknown pellet composition limited assessment of the effects of carbonates and/or lipids and precluded appropriate arithmetic correction of pellet δ^13^C values.

Despite the wide ranges around reported DTDF means above^14^, those means often have been (and continue to be) applied to field data in analyses such as mixing models, particularly when species-specific laboratory-derived DTDFs are lacking, with application of Δ^15^N = 3.4‰ and Δ^13^C = 0.4‰^10^ being particularly common^14^. However, inappropriate application of DTDFs can substantially affect results and interpretations of field studies. DTDFs are most commonly used in Bayesian mixing models (e.g. MixSIR, SIAR)^16,17^ which are highly sensitive to inaccurate DTDFs, producing highly erroneous estimates when DTDFs vary by > 1‰ and potentially less^14,43^. While many factors can influence DTDFs, including consumer physiological state, diet composition and quality, consumer size, age, and tissue composition^14^, recent studies have demonstrated a strong negative relationship between DTDFs and diet δ^15^N and δ^13^C values^14,15^. Our calculated DTDFs, while different from commonly applied mean values, were similar to those estimated from predictive equations (based on diet δ^15^N and δ^13^C values) in both Caut et al. (2009) and Hussey et al. (2014) (see Results). Yellowtail Δ^15^N_high_ and Δ^13^C_high_ estimates were also similar to those of Pacific bluefin tuna (*Thunnus orientalis*) (Δ^15^N = 1.9 ± 0.4‰; Δ^13^C = 1.8 ± 0.3) fed a similar diet of California Current-sourced squid and sardine^24^. This both corroborates the importance of diet δ^15^N and δ^13^C in determining DTDF and provides a slightly modified predictive equation (Results, Eqs. 1, 2), calculated from our two DTDFs, that can be applied to field-collected yellowtail data and potentially to other similar fish (e.g., large-bodied ectotherms) when species-specific estimates are not available.

Bayesian mixing models allow for multiple DTDFs to be entered as inputs. However, Caut et al. (2009) found that > 70% of studies used a single DTDF even when diet resources had substantially different δ^15^N and δ^13^C values. Our different DTDFs in the same yellowtail, when fed isotopically distinct diets, supports the assertion that a single DTDF is inappropriate in such cases. Based on our results, we recommend using our yellowtail-specific algorithm for field SIA data from yellowtail and potentially from similar fish. Since our algorithm is only calculated from the two DTDFs determined here, the equations from Caut et al. (2009) and Hussey et al. (2014) may also provide more robust and useful alternative and/or complementary DTDFs than static, mean DTDFs when applying Bayesian mixing model approaches to field data.

Yellowtail muscle turnover rates of ^15^N (*t*_0.5_ = 181 d) and ^13^C (*t*_0.5_ = 341 d) were higher (i.e., isotopic turnover in muscle took longer) than many values reported for teleosts, which have shown half-lives on the order of days to weeks (see literature syntheses^25–27^). While the incorporation of prey into consumer tissue and associated isotopic fractionation is a complex process, influenced by growth rate, thermoregulation, environment, diet quality, and nutrient routing, synthesis studies have shown a strong relationship between turnover rate and body size in fish^25–27^. However, most fish in captive turnover experiments are relatively small (< 1 kg, and often less than 100 g^24–26^). Our turnover rates were similar to larger-bodied (> 1 kg) teleosts and chondrichthyans (e.g. leopard shark *Triakis semifasciata*^44^; sandbar shark *Carcharhinus plumbeus*^45^; Pacific bluefin tuna^24^; gag grouper *Mycteroperca microlepis*^46^). In addition, our turnover rates were comparable to those that would be estimated from body size alone for vertebrate ectotherm muscle (~ 100–200 d)^26,27^, suggesting that captive yellowtail turnover dynamics were generally typical for ectothermic fish of their size, as well as the endothermic Pacific bluefin tuna.

Our results differed from the relatively consistent turnover rates of ^13^C and ^15^N in muscle that have been found across several taxa, and are predicted by metabolic scaling theory^26,27^. In contrast, we found slower turnover rates of yellowtail muscle ^13^C that were roughly 2 × those of ^15^N; similar dynamics were previously described in large-bodied (> 5 kg) Pacific bluefin tuna and sandbar sharks^24,45^. It is possible that this was partially driven by relative differences in yellowtail δ^13^C and δ^15^N endmember values, as the higher change of δ^15^N (~ 6‰) than δ^13^C (~ 1.5‰) (Fig. 2, 3) would likely drive more rapid change in ^15^N turnover in early stages of the experiment. Yellowtail were also fed a mixed fish/squid diet, and the possibility of intra-individual feeding variability (different proportions of fish and squid) and the different δ^13^C values of fish and squid (−19.7 and −17.9‰, respectively) could have driven different individual turnover rates and overall variability in turnover rates of δ^13^C. While several other mechanisms exist that potentially drive differences between ^13^C and ^15^N turnover rates in muscle (e.g., contributions of red blood cells, respiratory exchanges of C; Thomas and Crowther 2015), it is most likely that the relative contributions of growth and metabolic processes drive these differences in active, large-bodied fish. In larval and juvenile fish, muscle isotopic turnover is generally dominated by rapid growth^20,22,47^. This is well demonstrated in bluefin tuna; larval (< 1 g) Atlantic bluefin *Thunnus thynnus* showed a ^15^N half-life of 2.5 days with 100% turnover attributed to growth^48^, while sub-adult (> 4.5 kg) Pacific bluefin showed a ^15^N half-life of 167 days with 38% turnover attributed to growth^24^. In yellowtail here, growth contributed more to ^13^C turnover (84%) than to ^15^N turnover (44%), and growth was also found to contribute more to turnover of ^13^C (59%) than ^15^N (38%) in large-bodied Pacific bluefin^24^. Turnover rates of individual yellowtail provide additional insight to the relative importance of variable growth rates, as intra-specific differences in growth rate significantly affected turnover rates of both ^13^C and ^15^N (Fig. 5).

It is reasonable that as fish size increases and relative growth decreases, metabolic processes will play a greater role in isotopic turnover^49,50^. However, why this would differentially affect ^15^N and ^13^C is unclear, and likely includes metabolic dynamics of tissue protein turnover, routing of amino acids and lipids into muscle tissue proteins, and fractionation processes during catabolism. The isotopic dynamics of these processes are not well enough described or understood to assess their relative influence on ^15^N and ^13^C turnover^22,26,27^. While the processes underlying this result require further investigation, the implication is that δ^15^N and δ^13^C values of wild large-bodied fish should be considered independently, as δ^13^C values will represent longer timeframes of past resource use. This will influence both Bayesian mixing model diet reconstructions (as δ^13^C values will represent less current diet inputs than will δ^15^N) and isotopic clock approaches (that apply specific turnover rates of ^15^N and ^13^C to isotopic data to estimate time since diet shifts and/or migrations).

Though individual yellowtail growth rates in identical captive conditions varied (*W*_*r*_ = 2.7 ± 0.5; *k*ʹ = 0.0017 ± 0.0005 d^−1^), models of relative growth *W*_*r*_ versus change in δ^15^N and δ^13^C fit well (*r*^2^ = 0.96 and 0.91, respectively). The similarity of the growth required for 50% isotopic turnover (*G*_0.5_) for ^15^N and ^13^C (14% and 15%, respectively) suggests that turnover dynamics of both isotopes are similarly influenced by growth. Since time-based estimates of ^15^N turnover were substantially shorter than ^13^C, this reinforces the likelihood that metabolic processes play a more influential role in turnover of muscle ^15^N in yellowtail of this size. Model fits suggest that the relative growth in mass necessary for 95% turnover (*G*_0.95_) of ^15^N and ^13^C will occur with 77% and 82% increase in growth by mass, respectively (Table 4). Since relationships of age and growth, and length versus mass, have been described for California yellowtail^35,37^, these estimates can be scaled to larger or smaller yellowtail to estimate time-based estimates. In general, turnover rates will decrease with increasing yellowtail size due to the decrease in relative growth rates with size. For example, based on relationships in Baxter et al. (1960), the mass doubling times (*W*_*r*_ = 2) for a 1 kg, 2 kg, 4 kg, and 8 kg yellowtail are ~ 350, 550, 1000, and > 2000 d, respectively. These size-specific differences in relative growth will in turn change the time necessary for muscle isotopic turnover.

Substantially less growth was required for turnover of ^15^N and ^13^C than in similarly sized Pacific bluefin tuna with only slightly lower specific growth rates (0.0016 d^−1^) than yellowtail (0.0017 d^−1^), as 72% and 209% increases in growth were required for 50% turnover of ^15^N and ^13^C, respectively, in Pacific bluefin tuna muscle^24^. This again indicates the relative importance of metabolic processes in turnover of muscle ^15^N and ^13^C in active, large-bodied fish. As regional endotherms, Pacific bluefin tuna have higher internal temperatures and consequently higher metabolic demands than ectotherms^51^, presumably leading to increased rates of the metabolic functions that influence turnover of ^13^C and ^15^N. As such, growth-based turnover estimates here may be more appropriately applied to other ectothermic teleosts, while those from Pacific bluefin may be more appropriate for other endothermic fish (*e.g*., other *Thunnus* species, lamnid sharks, opah *Lampris* spp).

Our results lend insight into the application of various laboratory-derived isotopic parameters to wild fish. First, our different DTDFs from diets of different isotopic composition strongly support other studies that call for diet-based DTDFs^14,15^. Caut et al. (2009) found that > 33% of studies used Δ^15^N and Δ^13^C values that were > 2‰ different than estimated diet-dependent discrimination factors (DDDFs), which would render mixing model results highly misleading. In the absence of specific DTDFs for the study species, estimated DDDFs from algorithms provided in Caut et al. (2009) and Hussey et al. (2014) may provide a useful starting point.

Second, the variability of δ^15^N and δ^13^C observed at the start, middle, and end of the diet switch experiment suggests that intra-specific variability may be lower for end-member isotopic parameters (DTDFs) than mid-member parameters (turnover rates). Accordingly, applied DTDFs will be more reliable than turnover rates in isotopic clock approaches. Isotopic clocks have been applied to large active teleosts with some success^31^; in one study, an isotopic clock applied to Pacific bluefin tuna suggested two ‘pulses’ of arrival in a new isotopic environment^34^, and these results were later corroborated by an extensive electronic tagging study of juvenile bluefin^52^. However, that isotopic clock used a bootstrapping technique incorporating the error around turnover rates, was applied to a large sample size (*n* = 428 individuals), and showed wide variability of arrival time estimates around peak arrival times^34^. Consequently, application of an isotopic clock to one or several individuals may yield highly inaccurate results depending on prior isotopic turnover rates in those specific individuals (which cannot be measured). As such, we suggest that the turnover rates here be used in analytical frameworks that incorporate uncertainty and, if possible, be applied to large datasets.

Third, an ongoing question with Bayesian mixing model diet reconstructions is the time required for consumers, and mixing model estimates, to reflect current feeding conditions. Our application of the Bayesian mixing model MixSIR to yellowtail feeding on fish/squid showed that ~ 520 days were required to reflect this diet. However, contrary to expectations, this timeframe did not require 3–4 half-life periods for both isotopes and was not limited by the isotope with slower turnover (^13^C), which would have required > 1000 days (≥ 3 half-lives of ^13^C) to reflect current diet. The limiting isotope was that with more rapid turnover (^15^N), and accurate mixing model estimates required ~ 3 half-life periods of that isotope. Thus while mixing model applications must continue to consider the prior timeframes represented by diet estimate outputs, estimates may require less time to reflect current feeding than expectations from turnover rates alone. Further analyses using simulated or lab-derived data will further clarify these dynamics to constrain the timeframes represented by mixing model outputs.

The challenges of rearing active, fast-growing teleosts in captivity while tracking diet and isotopic turnover make fully controlled conditions difficult to maintain. In this study, diet type, quantity, and ratios were maintained, but seawater temperature and salinity were dependent on local conditions (though temperature was monitored, and relatively constant at ~ 18 °C). Individual fish grew at different rates despite uniform rearing conditions; while variable fish size may have influenced turnover rates and DTDFs (due to physiological changes during growth), it also allowed for investigation of the relationship of growth and turnover rates (Fig. 4). DTDF estimates are also complicated by the inability to fully characterize the nutritional and isotopic composition of ingested prey. In particular, the uncertainty of biological sources of commercial pellet feed, which were unavailable, and the high C:N of pellet feed suggests that DTDFs from this diet should be interpreted with caution. Analysis of fish and squid muscle excludes other ingested parts (*e.g*., bones containing calcium carbonate). However, field studies with omnivorous animals are subject to the same uncertainty due to mixed diet, making the uncertainty around these reported estimates appropriate for field-collected data. Tracking turnover in individual fish provided some quantification of expected uncertainty from a mixed diet, but the parameters provided here should be interpreted and applied with the same considerations of uncertainty and variability that are inherent to all isotopic ecology studies.

Laboratory studies using diet switches with captive animals provide species-specific isotopic parameters that aid interpretation of wild data. Our calculated yellowtail DTDFs corroborate diet-dependence of DTDFs and the importance of applying multiple DTDFs in mixing model approaches. Turnover rates demonstrated that intra-specific variability of isotopic turnover can be substantial, contributing to the error reported around turnover rate estimates. While quantitative framework now exists for predicting DTDFs based on diet and turnover rates based on body size, the variability in those published relationships and the variation we observed here show that species- and lifestage-specific studies still provide the most precise isotopic parameters to ecologists for targeted species of study, especially for large-bodied animals for which fewer data exist. Future studies will both expand the breadth of species for which important isotopic parameters are necessary, refining models that allow for estimates of DTDFs and isotopic turnover based on animal type, diet, body size, and lifestage. This will continue to improve interpretations of field data using stable isotope approaches.

## Materials and methods

### Ethics statement

All procedures used in these experiments were in accordance with protocol SW1401 of the SWFSC Animal Care and Use Committee. YOY California yellowtail were collected under California Department of Fish and Wildlife Scientific Collection Permit #SC-12372. Experimental protocols were approved by the National Oceanic & Atmospheric Administration Southwest Fisheries Science Center (NOAA SWFSC) Animal Care & Use Committee. Reporting of methods and results were in compliance with ARRIVE guidelines for animal research^53^.

### Collection and captive husbandry of yellowtail

YOY California yellowtail (14–19 cm) were collected from offshore floating kelp mats near San Diego, CA, USA on September 12, 2012. Fish were caught using unbaited sabiki bait rigs, then immediately transferred to an onboard flow-through holding tank. On land, fish were transported in the holding tank on the trailered vessel to NOAA’s SWFSC Experimental Aquaria Facility in La Jolla, CA where they were transferred via dipnet to holding tanks. Fish were first held in 300 × 150 × 90 cm oval tanks (~ 3200 L) with flow-through, filtered local seawater at local ambient seawater temperature (~ 18 °C) and reared on a diet of Bio-Oregon BioTrout feed pellets. Pellets were presented immediately to newly transferred fish and fish began feeding 0–7 days after capture. Yellowtail were fed pellets 6 days/week to apparent satiation. After 525 days, the now larger yellowtail (42–50 cm) were transferred to a larger circular tank (diameter 3.7 m, capacity 9600 L) with the same filtered seawater at the same ambient temperatures (~ 18 °C) and tagged in the dorsal musculature with uniquely numbered and colored Floy plastic spaghetti tags. Yellowtail were switched to a diet of Pacific mackerel (*Scomber japonicus*) and market squid (*Doryteuthis opalescens*), both sourced off the coast of southern California (McRoberts Sales Co.). Yellowtail were fed mackerel and squid (by mass: 62% mackerel, 38% squid) 6 days/week to apparent satiation.

Yellowtail muscle was sampled at *t* = 0 (the day of diet switch), followed by sampling intervals ranging from 27 to 119 days (mean interval 60 ± 27 days) depending on perceived condition of yellowtail and conditions for sampling. Samples were collected by lowering tank water levels, capturing yellowtail in a rubber knotless net, then transferring fish to vinyl cradles. Biopsy punches (Cook Quick-Core G07821) were used to remove 0.1–0.2 g of white muscle from the dorsal musculature. When possible, fork length (FL; cm) was measured at the time of sampling. Muscle tissue was also collected from the diet (dorsal musculature from mackerel and mantle tissue, with the outer membrane removed, from squid) throughout the study for SIA. Sampling continued until yellowtail were removed due to poor condition, suffered natural mortality, or became too large to remain in the holding tank (t = 753 days after diet switch, the endpoint of the study).

YOY yellowtail (13.7–18.8 cm, 15.7 cm ± 1.7; 0.02–0.08 kg; 0.04 kg ± 0.02) fed well on pellet diet, increasing ~ threefold in length and ~ 40-fold in mass over 525 days. After 525 days, 21 similar-sized (42–50 cm FL, 46.0 cm ± 2.5; 1.0–2.2 kg, 1.7 kg ± 0.3) individual yellowtail were selected for the diet switch experiment to fish/squid (Fig. 1). During the course of the diet switch experiment, 11 individual fish were removed from captive conditions before reaching apparent isotopic steady-state due to poor physical condition, lack of feeding, or natural mortality. A total of 10 yellowtail fed consistently in captivity for a long enough period to reach apparent steady-state with new diet (595–753 days) allowing calculation of individual turnover rates in these fish. This allowed for estimates of Δ^15^N_low_ and Δ^13^C_low_ and population-wide isotopic turnover estimates from 21 fish subjected to the diet switch, and Δ^15^N_high_ and Δ^13^C_high_ and individual yellowtail turnover estimates from 10 individuals in captive conditions for 595–753 days that reached steady-state with new diet.

### SIA of yellowtail and diet

Yellowtail dorsal muscle tissue and prey muscle tissue samples were immediately stored in cryovials at  − 20 °C. Feed pellets were analyzed whole. All samples were then frozen at  − 80 °C and subsequently lyophilized and ground to a homogenous powder for isotope analysis. The δ^13^C and δ^15^N values of all samples were determined at the University of Hawaii using an on-line C–N analyzer coupled with a Delta XP isotope ratio mass spectrometer. Replicate reference materials of atmospheric nitrogen and V-PDB were analyzed every 10 samples, and analytical precision was < 0.2‰ for δ^13^C and δ^15^N. Isotope ratios are described by:

3 $$\delta^{q} X = \, \left( {R_{A} /R_{{{\text{standard}}}} - \, 1} \right) \, \times \, 1000,$$

where *q* is the isotope of interest, *X* is the element of interest, *R*_A_ is the ratio of the rare to the common isotope, and *R*_standard_ is the isotope standard Air or V-PDB. Isotope values are reported as per mille (‰).

### Arithmetic corrections of δ^13^C values

While both chemical and arithmetic lipid extractions have been shown to be effective methods to correct bias in δ^13^C values due to lipid-content, arithmetic corrections preserve sample integrity and simplify sample preparation^54^. Since studies have noted effects of chemical lipid extraction on δ^15^N^54–56^ and suggested separate treatment for δ^15^N and δ^13^C analyses, and biopsy samples did not always provide adequate material for such treatment (especially from smaller yellowtail), we chose to arithmetically correct for lipid content. δ^13^C values of yellowtail muscle and diet items (mackerel and squid) were arithmetically lipid-normalized based on mass C:N ratios using muscle- and organism type-specific lipid normalization algorithms^55^. For yellowtail and mackerel, we used a muscle-specific lipid correction algorithm derived from a suite of fish species:

4 $${\updelta }^{13} {\text{C}}^{\prime } \, = P{-} \, \left( {P*F/{\text{ C}}:{\text{N}}} \right) \, + {\updelta }^{13} {\text{C}}_{{{\text{tissue}}}}$$

where δ^13^C' is the arithmetically-corrected δ^13^C value, C:N is the C/N ratio by mass of the specific sample, and *P* and *F* are parameter constants based on measurements by Logan et al.^55^. For squid, we used the same equation, with parameters *P* and* F* derived from invertebrates which included shortfin squid *Illex illecebrosus*. Relatively low C:N ratios in squid (3.4 ± 0) led to minimal differences in δ^13^C and δ^13^C' in squid. Since pellet composition was unknown and no appropriate arithmetic δ^13^C correction was available, bulk δ^13^C values are reported for pellets.

### Calculating DTDF

We calculated two DTDFs (Δ^15^N and Δ^13^C), one for pellet diet and one for fish/squid diet. For both DTDFs, Δ^15^N and Δ^13^C were calculated from the mean difference between yellowtail muscle and respective diet δ^15^N and δ^13^C values when yellowtail were at isotopic steady-state. Δ^15^N_low_ and Δ^13^C_low_ were calculated before the diet switch, after yellowtail fed on pellets for 525 days. Δ^15^N_high_ and Δ^13^C_high_ were calculated after yellowtail that had reached steady-state with the fish/squid diet using the weighted (by proportion mass in diet) mean δ^15^N and δ^13^C values of fish/squid diet. DTDF values were calculated according to the equation:

5 $$\Delta_{{{\text{diet}} }} = {\text{ mean }}({\updelta }_{{{\text{yellowtail}}}} {-}{\updelta }_{{{\text{diet}}}} )$$

where Δ_diet_ represents the diet- (‘low’ or ‘high’) and isotope-specific DTDF, δ_yellowtail_ is the δ^15^N or δ^13^C value of yellowtail that reached steady-state with diet, and δ_diet_ is the mean δ^15^N or δ^13^C value of the food (pellet or fish/squid). For the fish/squid diet, δ^13^C values were arithmetically lipid-corrected^55^ and weighted by the proportional mass of each item in the diet (62% and 38%, respectively). For Δ^15^N_low_ and Δ^13^C_low_, we assumed YOY yellowtail had reached steady-state with pellet diet after 525 days. This was supported by the length of time (525 days) and relative growth (mass_final_/mass_initial_ =  ~ 2 kg/0.05 kg =  ~ 40) of yellowtail on pellet feed, both of which are substantially higher than what is demonstrably necessary for small fish to reach steady-state with diet.

Since we calculated two different DTDFs that were diet-dependent, as has been previously demonstrated^14,15^, we compared our experimentally-derived DTDF values to the diet-dependent DTDF algorithms reported by Caut et al. 2009 (Δ^15^N and Δ^13^C) and Hussey et al. 2014 (Δ^15^N only), both of which used DTDFs for fish to derive linear equations for diet-based estimates of DTDFs. We used the fish white muscle equation from Caut et al. 2009 for Δ^15^N and Δ^13^C:

6 $$\Delta^{15} {\text{N}} = \, - 0.281({\updelta }^{15} {\text{N}}_{{{\text{diet}}}} ) \, + \, 5.879$$

7 $$\Delta^{13} {\text{C}} = \, - 0.248({\updelta }^{13} {\text{C}}_{{{\text{diet}}}} ) \, {-} \, 3.477$$

and the fish (muscle and/or whole) equation from Hussey et al. 2014 for Δ^15^N:

8 $$\Delta^{15} {\text{N}} = \, - 0.27({\updelta }^{15} {\text{N}}_{{{\text{diet}}}} ) \, + \, 5.92$$

and compared those estimated DTDFs to our experimentally-derived values.

### Time-based isotopic turnover

Sequential sampling of individual yellowtail allowed for quantification of turnover rate in (1) all yellowtail that reached steady-state with new diet and (2) the pooled population of captive yellowtail. We used exponential fit models and to quantify yellowtail muscle tissue turnover rate of δ^13^C and δ^15^N, as used previously^24,57–60^:

9 $$\delta_{t} = ae^{ - \lambda t} + \, c,$$

where *δ*_*t*_ is the stable isotope value at time *t*, *a* and *c* are parameters derived from the best fit, and λ is a data-derived first-order rate constant. Parameters *a* and *c* represent specific parameters: *a* = difference (‰) between initial and final steady-state values and *c* is the model-estimated final isotope steady-state value^24,60^. The isotope-specific half-life (*t*_0.5_) was then calculated:

10 $$t_{0.5} = \, \ln \left( 2 \right)/\lambda$$

for different *λ* values derived for δ^15^N and δ^13^C, for both individual yellowtail and the grouped population. We used a modified equation from Buchheister and Latour^21^ to calculate the time needed to obtain a given percentage (*α*) of complete turnover:

11 $$t_{\alpha /100} = \, \ln \left( {1 - \alpha /100} \right)/\lambda$$

where *t*_α/100_ is the time needed to attain *α*% turnover and *λ* is the data-derived first-order rate constant.

### Growth-based isotopic turnover

Fish length (fork length or FL; cm) was recorded at *t*_0_, various time steps throughout the experiment concurrent with tissue sampling, and *t*_f_. Direct measurements of yellowtail mass (kg) were taken at *t*_0_. Some mass measurements were taken with FL during the experiment; however direct measurements of mass during sampling were not always possible due to the difficulty of weighing large, active fish and the priority to minimize stress on captive fish during sampling events. When only length was available for individual yellowtail at specific timesteps, mass at time *t* (*W*_*t*_) was estimated using the length–weight equation from Baxter 1960:

12 $$W_{t} = \, 7.747 \, \times \, 10^{ - 8} \left( {{\text{FL}}_{t} {\text{mm}}} \right)^{2.84}$$

Relative gain in mass (*W*_*R*_, hereafter referred to as ‘relative growth’) was then calculated:

13 $$W_{R} = W_{f} /W_{i}$$

where *W*_*f*_ is the measured final mass and *W*_*i*_ is the initial mass estimate from SL. Using the equation from Ricker^61^ for *W*_*f*_:

14 $$W_{f} = W_{i} e^{k^{\prime}t}$$

where *k'* is the group specific growth-rate constant, we derive *k'*:

15 $$k^{\prime } = \, \ln \left( {W_{R} } \right)/t$$

and can obtain the growth rate constant *k'* for individual fish using relative growth (*W*_*R*_) and time in captivity *t*. Hesslein et al.^62^ describes the isotope value of a fish at time *t* (*δ*_*t*_) as:

16 $$\delta_{t} = \delta_{f} + (\delta_{i} {-}\delta_{f} )e^{{ - }{\left( {k^{\prime} \, + \, m} \right)t}}$$

where δ_*f*_ is the final isotope value at steady-state with diet, δ_*i*_ is the initial isotope value before the diet switch, *m* is the metabolic turnover constant. This is a modification of Eq. (3), where δ_*f*_ = *c*, (δ_*i*_ – δ_*f*_) = *a*, and (*k'* + *m*) = *λ*. Thus we calculate *λ* from Eq. (3), *k*' from Eq. (13), and use Eq. (14) to calculate the metabolic constant *m* based on turnover rates of δ^15^N and δ^13^C^24^. We can also calculate the amount of relative growth needed to achieve α percent turnover of δ^13^C and δ^15^N^21,24^:

17 $$G_{\alpha /100} = \, \exp \, \left( {\ln \left( {1 \, {-}\alpha /100} \right)/\lambda } \right)$$

and growth-based turnover can be calculated:

18 $$G_{0.5} = \, \exp \, \left( {\ln \left( {0.5} \right)/\lambda } \right)$$

where *G*_0.5_ is the growth-based half-life and *λ* is the data-derived rate constant from growth-based model fits to yellowtail muscle δ^13^C and δ^15^N. We estimated the proportion of isotopic turnover due to growth (*P*_*g*_) and the proportion of turnover due to metabolism (*P*_*m*_) as the proportion of *k'* and *m*, respectively, of the overall isotopic turnover constant λ^21,24,61^:

19 $$P_{g} = k^{\prime } /\lambda$$

20 $$P_{m} = \, m/\lambda$$

We applied Eqs. 13–20 to δ^13^C and δ^15^N values in yellowtail muscle tissue and report growth turnover rate constants and overall estimated contribution of growth and turnover to observed δ^15^N and δ^13^C turnover in captive yellowtail.

### Applicability to field data

Typical applications of δ^13^C and δ^15^N data from field-collected animals include approaches that assume isotopic steady-state with diet (e.g. Bayesian mixing models) and approaches that utilize isotopic values after an assumed or inferred shift in diet and/or habitat (e.g. isotopic clock approaches). Sequential sampling of individual yellowtail allowed for investigation of variability in δ^13^C and δ^15^N values at steady-state with pellet feed, at various time intervals after a diet switch, and at steady-state with fish/squid diet. Sequential sampling reduced the influence of individual variability in assessment of isotopic parameters. We compared time after diet switch to three metrics of variability (variance, range, standard deviation) of δ^13^C and δ^15^N throughout the experimental period to assess the robustness of approaches assuming steady-state and/or changing δ^13^C and δ^15^N values in wild predators.

Since tissue δ^13^C and δ^15^N values represent a time-integrated signature of prior feeding, Bayesian mixing model approaches represent both prior and current diet until consumers reach steady-state. To allow comparison of isotopic turnover rates and the time required for Bayesian mixing models to adequately represent current diet after a diet switch, we applied the Bayesian mixing model MixSIR^17^ to all timesteps of the diet switch experiment. For inputs, we used δ^13^C and δ^15^N from sampled yellowtail, two different DTDFs calculated here (one for pellet feed, one for fish/squid; see Results), and mean δ^13^C and δ^15^N values of pellets and fish/squid as diet inputs. For each timestep we ran 10^4^ iterations and uninformative priors. Mixing model outputs allowed for estimation of the time required for the model to represent the actual current diet of yellowtail following the diet switch (100% fish/squid).
